# Characteristics and survival of primary urothelial carcinoma of the prostate: A multi-center retrospective study of 18 cases

**DOI:** 10.1016/j.neo.2023.100961

**Published:** 2023-12-23

**Authors:** Junjie Ji, Tian Liu, Yu Yao, Wen Liu, Hao Ning, Tongyu Wang, Guiming Zhang

**Affiliations:** a Department of Urology, The Affiliated Hospital of Qingdao University, Qingdao, China; b Department of Urology, Beijing Hospital, Beijing, China; c Department of Urology, Shandong Provincial Hospital, Jinan, China; d Department of Radiology, The Affiliated Hospital of Qingdao University, Qingdao, China

**Keywords:** Primary urothelial carcinoma of the prostate, Prostate cancer, Urothelial carcinoma, Prognosis, Multicenter

## Abstract

**Objectives:**

To explore the features, treatment, and outcomes of primary urothelial carcinoma of the prostate (PUCP) in a multicenter study.

**Methods:**

The clinical and imaging features, pathological findings, treatment, and outcomes of patients diagnosed with PUCP from January 2011 to April 2022 at three institutions were collected and analyzed. The Kaplan–Meier method and log-rank test were used to assess survival rates of the overall group and survival differences between groups according to TNM stage.

**Results:**

The study cohort comprised 18 patients with PUCP of mean age 72.4±7.8 years. Dysuria and urinary frequency were the most common symptoms (77.8 %). Sixteen (88.9 %) patients had normal serum total PSA concentrations. Most patients showed abnormalities on urinalysis. MRI was the most accurate diagnostic imaging method (88.9 %). As to immunohistochemistry findings, GATA-3 (81.8 %) and P63 (84.6 %) were positive in most examined patients; however, no lesions were positive for PSA. Three (17.6 %) patients with T1N0M0 and T2N0M0 tumors underwent radical cystectomy. Eleven (64.7 %) patients which almost all had T4 tumors received systematic therapy, most of them receiving chemotherapy with gemcitabine and cisplatin, and radiotherapy. The median overall survival was 42 months, and the median progression-free survival 25 months, the latter being significantly longer in patients with T1–2 than in those with T3–4 disease (p=0.035).

**Conclusion:**

PUCP, a rare but highly aggressive type of prostate cancer, should be considered in men with abnormalities on MRI and normal serum PSA concentrations. Positive GATA-3, P63, and negative PSA are typical immunohistochemistry features. Radical cystectomy and systematic therapies can be effective.

## Introduction

According to 2020 global cancer statistics, prostate cancer is the second most common malignancy after lung cancer in men worldwide, over 90 % of which are prostatic adenocarcinoma [1]. Besides the urothelium of the urinary tract, urothelial carcinoma can also originate from prostatic stroma, prostatic ducts, and the mucosa of prostatic transitional urothelium, the latter being defined as primary urothelial carcinoma of the prostate (PUCP) [2]. PUCP is extremely rare, reportedly accounting for approximately 1 % to 5 % of prostatic malignancies [3,4].

Since PUCP was first described as Bowen's disease in 1952 [5], only limited data on this type of tumor have been published, nearly all as case reports. Pathology and immunohistochemistry are the main diagnostic modalities. Several immunohistochemical markers have been reported to aid diagnosis, including GATA-3, P63, and high-molecular weight cytokeratin [6,7]. Because few patients with PUCP have high serum PSA concentrations [8,9], the opportunity for early diagnosis is missed in most patients. This, together with the characteristic extreme aggressiveness and strong tendency to invasion, means that most patients have evidence of local progression or distant metastasis at the time of diagnosis [10].

Considering its poor prognosis, a general understanding drawn from studying numbers of patients with this disease from multiple institutions is important. In the present study, we explored the features, treatment, and outcomes of PUCP in patients from multiple centers to provide insights into this disease.

## Material and methods

### Study cohort

We retrospectively selected patients who were pathologically diagnosed with PUCP between January 2011 and April 2022 in the Affiliated Hospital of Qingdao University, Beijing Hospital, and Shandong Provincial Hospital Affiliated to Shandong First Medical University. Patients with a history of bladder or urethral urothelial carcinoma were excluded to guarantee that the urothelial carcinoma had originated in the prostate.

### Characteristics of patients

We collected relevant data for the selected PUCP patients, including age, body mass index, initial symptoms, results of DRE, histories of hypertension, diabetes, drinking, and smoking, serum tumor marker concentrations, results of urinalysis, imaging findings, diagnostic modalities, histopathology, immunohistochemistry, TNM stage, and treatment. All patients were followed up until March 2023 or death.

### Statistical analyses

The collected clinical information was analyzed as descriptive statistics such as proportion. Overall survival (OS) was defined as time from the date of pathologic diagnosis to the time of last follow-up or death from any cause. Progression-free survival (PFS) was defined as the time from the date of pathologic diagnosis to clinical progression or death from any cause. The Kaplan–Meier method was used to assess OS and PFS. We further analyzed differences in OS and PFS between patients with different TNM stage disease according to AJCC stage. The log-rank test was used to analyze survival differences among these variables. Two-sided P-values <0.05 were considered to denote significant differences in all statistical tests. Statistical analyses and graphics were performed using R software (version 4.1.0).

## Results

### Clinical characteristics

The study cohort comprised 18 patients with pathological diagnoses of PUCP from January 2011 to April 2022, including seven in the Affiliated Hospital of Qingdao University, seven in Beijing Hospital, and four in Shandong Provincial Hospital Affiliated to Shandong First Medical University. These patient's characteristics are listed in Table 1 and their relevant personal, clinical, and immunohistochemical data are summarized in Table 2. Twelve (66.7 %) patients had T4 disease, nine (50.0 %) N1, and five (27.8 %) patients M1 at the time of diagnosis.

**Table 1:** Characteristics and follow-up of included cases.

No.	Age	BMI	Hypertension	Diabetes	Drinking	Smoking	Diagnostic treatment	Histopathology	Staging	Treatment	Follow-up
1	70	31.74	No	No	Yes	No	TURP	High-grade urothelial carcinoma & adenocarcinoma	T2N0M0	Gemcitabine for systemic chemotherapy	Alive with no progression for 18 months
2	85	19.95	No	No	No	No	TURP	High-grade urothelial carcinoma	T4N0M0	Goserelin for systemic hormonal treatment	Progressed 6 months and died 31 months after diagnosis
3	59	25.95	No	No	No	No	Prostate needle biopsy	High-grade urothelial carcinoma	T4N1M1	NA	NA
4	73	29.41	Yes	No	No	No	TURP	High-grade urothelial carcinoma	T1N0M0	Radical cystectomy and bilateral lymphadenectomy	Progressed 3 months and died 42 months after diagnosis
5	62	28.06	Yes	Yes	Yes	Yes	TURP	High-grade urothelial carcinoma	T4N0M0	Gemcitabine plus cisplatin and docetaxel for systemic chemotherapy, radiation therapy	Progressed 2 months after diagnosis and alive for 51 months
6	66	19.72	No	No	No	Yes	Prostate needle biopsy	High-grade urothelial carcinoma	T4N1M1	Gemcitabine for systemic chemotherapy	Died 2 months after diagnosis
7	83	22.86	Yes	No	No	No	TURP	High-grade urothelial carcinoma	T1N0M0	No treatment	Alive with no progression for 11 months
8	70	26.73	No	Yes	No	No	TURP	High-grade urothelial carcinoma	T4N1M0	Radical prostatectomy and bilateral lymphadenectomy, radiation therapy	Progressed 2 months and died 8 months after diagnosis
9	70	23.45	Yes	No	No	No	TURP	High-grade urothelial carcinoma	T4N1M1	Gemcitabine plus cisplatin for systemic chemotherapy, radiation therapy	Progressed 5 months and died 104 months after diagnosis
10	76	26.22	Yes	No	No	No	TURP	High-grade urothelial carcinoma	T4N1M0	Goserelin for systematic hormonal treatment	Alive with no progression for 47 months
11	66	28.41	Yes	Yes	Yes	Yes	Prostate needle biopsy	High-grade urothelial carcinoma	T4N1M1	Disitamab Vedotin plus Tislelizumab for targeted therapy plus immunotherapy, radiation therapy	Alive with no progression for 12 months
12	67	30.42	No	No	Yes	Yes	Prostate needle biopsy	High-grade urothelial carcinoma	T4N1M0	Gemcitabine combined cisplatin for systemic chemotherapy, TURP	Died 37 months after diagnosis
13	88	24.77	No	No	No	No	TURP	High-grade urothelial carcinoma	T4N1M0	Gemcitabine for systemic chemotherapy, percutaneous nephrostomy	Progressed 3 months and died 46 months after diagnosis
14	71	20.07	No	No	No	Yes	TURP	High-grade urothelial carcinoma	T1N0M0	No treatment	Progressed 80 months after diagnosis and alive for 85 months
15	69	27.04	Yes	Yes	No	Yes	Prostate needle biopsy	High-grade urothelial carcinoma	T4N1M1	Gemcitabine plus cisplatin for systemic chemotherapy, radiation therapy	Progressed 8 months and died 15 months after diagnosis
16	79	21.97	No	No	No	No	TURP	High-grade urothelial carcinoma	T4N0M0	Gemcitabine plus cisplatin for systemic chemotherapy, radiation therapy	Died 25 months after diagnosis
17	78	24.84	No	Yes	No	No	TURP	High-grade urothelial carcinoma	T2N0M0	Radical cystectomy and bilateral lymphadenectomy	Alive with no progression for 41 months
18	71	23.31	Yes	Yes	No	Yes	TURP	High-grade urothelial carcinoma	T1N0M0	Radical cystectomy and bilateral lymphadenectomy	Alive with no progression for 21 months

BMI, body mass index; TURP, transurethral prostatectomy

**Table 2:** Clinical and immunohistochemical findings at diagnosis.

Characteristics	Percentage (no.)
**Age**	72.4 ± 7.8
**BMI**	25.3 ± 3.6
**T stage**	
T1	22.2 % (4/18)
T2	11.1 % (2/18)
T3	0 % (0/18)
T4	66.7 % (12/18)
**N stage**	
N0	50.0 % (9/18)
N1	50.0 % (9/18)
**M stage**	
M0	72.2 % (13/18)
M1	27.8 % (5/18)
**Symptoms**	
Dysuria	77.8 % (14/18)
Urinary frequency	77.8 % (14/18)
Pain in urination	33.3 % (6/18)
Hematuria	38.9 % (7/18)
**Physical examination**	
Abnormal DRE	36.4 % (4/11)
**Serum tumor markers**	
Increased TPSA	11.1 % (2/18)
Increased PSAD	0 % (0/18)
Increased PAP	33.3 % (2/6)
**Urinalysis**	
Positive urinary erythrocyte	88.2 % (15/17)
Positive urinary leukocyte	70.6 % (12/17)
Positive urinary protein	88.2 % (15/17)
**Imaging examination**	
Abnormality in B-ultrasound examination	55.6 % (10/18)
Abnormality in MRI	88.9 % (8/9)
Abnormality in enhanced CT	50.0 % (2/4)
Abnormality in whole body bone imaging	28.6 % (2/7)
**Immunohistochemistry**	
Positive PSA	0 % (0/17)
Positive PSAP	0 % (0/3)
Positive PSMA	0 % (0/2)
Positive CK7	76.9 % (10/13)
Positive CK20	61.5 % (8/13)
Positive CK34βE12	50.0 % (3/6)
Positive GATA-3	81.8 % (9/11)
Positive P504S	55.6 % (5/9)
Positive P63	84.6 % (11/13)
Positive AMCAR	46.2 % (6/13)

DRE, digital rectal exam; TPSA, total prostate specific antigen; PSAD, prostate specific antigen density; PAP, prostatic acid phosphatase; MRI, magnetic resonance imaging; CT, computed tomography

The mean age of included patients was 72.4±7.8 years and the mean body mass index 25.3±3.6. Dysuria and urinary frequency were the most common symptoms of PUCP, each of these being reported in 14 (77.8 %) patients. Seven (38.9 %) patients had gross hematuria, and six (33.3 %) presented with painful urination. Eleven patients underwent DRE, abnormal nodules being detected in four (36.4 %) of them. Eight (44.4 %) patients had a history of hypertension, six (33.3 %) a history of diabetes, four (22.2 %) a history of drinking, and seven (38.9 %) a history of smoking.

### Laboratory and imaging findings

Serum tumor markers were examined in all patients. However, only two (11.1 %) had high total PSA concentrations and none (0 %) had increased PSA density. Further, six patients were tested for serum prostatic acid phosphatase; rising PAP was found in only two (33.3 %) of them. In contrast, most patients showed abnormalities on urinalysis. One patient did not undergo urinalysis. Urine was positive for erythrocytes in 15 (88.2 %) patients, for leukocytes in 12 (70.6 %), and for protein in 15 (88.2 %).

B-ultrasound was the most commonly performed imaging examination (18 of 18); however, it detected tumors in only 10 (55.6 %) of the 18 patients. In B-ultrasound examinations, the tumors presented with low or mixed signals and unclear borders. Similarly, tumors were detected in only two of four patients who underwent enhanced lower abdominal CT or CT urography. Both were stage IV tumors and showed as irregular masses with heterogeneous density and uneven enhancement. Nine patients underwent MRI, which detected tumors in eight (88.9 %) of them. The tumors appeared as irregular low-signal lesions in T2-weighted images and irregular hyperintense foci in diffusion-weighted images. Additionally, whole body bone imaging revealed bone metastases in two (28.6 %) of seven patients.

### Pathology and immunohistochemistry

**Fig. 1:**
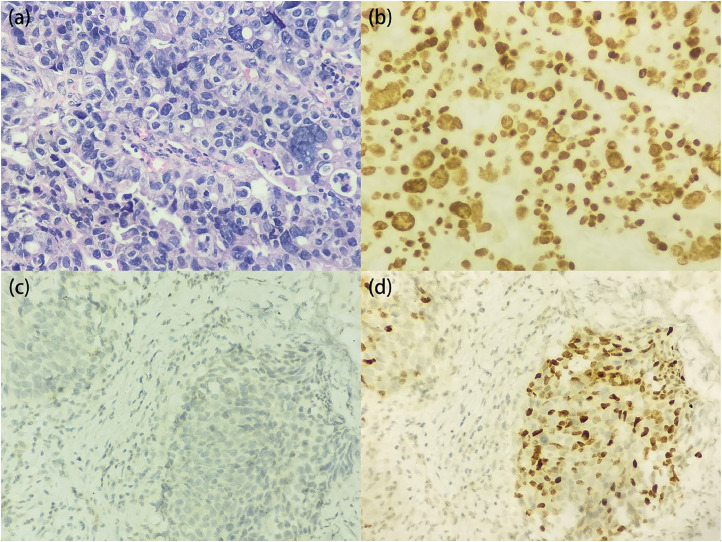
Photomicrographs of PUCP samples. (a) Hematoxylin and eosin stained section (magnification: ×400). Immunohistochemistry revealed that the tumor cells were negative for PSA (c, magnification: ×400), but positive for GATA-3 (b, magnification: ×400) and P63 (d, magnification: ×400). PSA, prostate specific antigen; PUCP, primary urothelial carcinoma of the prostate.

PUCP was pathologically confirmed in all study patients, pathological diagnoses being made on tissue obtained by TURP in 13 (72.2 %) patients and by needle biopsy of the prostate in five (27.8 %) patients. A representative hematoxylin and eosin stained section is shown in Fig. 1a. The results of immunohistochemistry are expressed as “number positive/total tested” and were as follows: GATA-3 (9/11) (Fig. 1b), PSA (0/17) (Fig. 1c), prostate specific acid phosphatase (0/3), prostate-specific membrane antigen (0/2), P63 (11/13) (Fig. 1d), CK7 (10/13), CK20 (8/13), CK34βE12 (3/6), P504S (5/9), and alpha methyl acyl CoA racemase (6/13). Considering above results, all patients were high-grade urothelial carcinoma.

### Treatment and prognosis

Information on treatment was not available for one patient. Of the remaining patients, two (11.8 %) with T1N0M0 tumors received no treatment after a pathological diagnosis had been made, three (17.6 %) with T1–2N0M0 tumors underwent radical cystectomy and bilateral lymphadenectomy alone, one (5.9 %) with a T4N1M0 tumor underwent radical prostatectomy plus bilateral lymphadenectomy and adjuvant radiotherapy, and the remaining 11 (64.7 %) received systematic therapy. Of the 11 patients who received systematic therapy, three received gemcitabine plus cisplatin combined with radiotherapy, three received gemcitabine monotherapy, two received goserelin as systematic hormonal treatment, one received gemcitabine, cisplatin, and docetaxel combined with radiotherapy, one received gemcitabine combined with cisplatin, and one with a T4N1M1 tumor received targeted therapy combined with immunotherapy and radiotherapy.

**Fig. 2:**
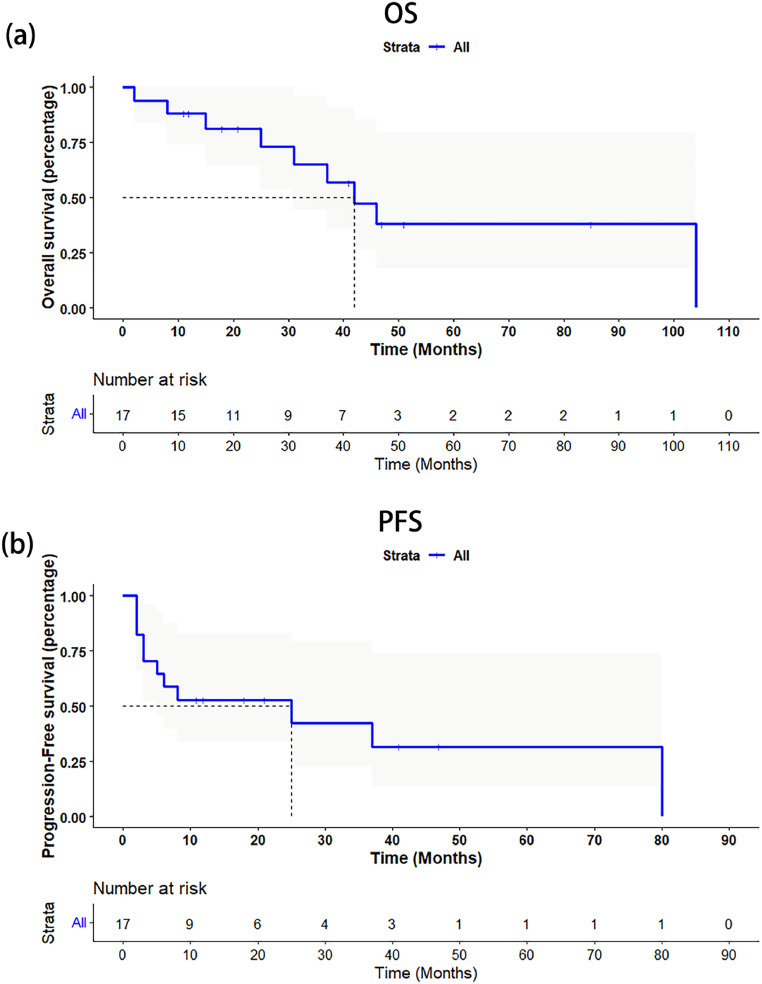
Kaplan–Meier curves of survival in whole cohort. (a) Overall survival. (b) Progression-free survival.

**Fig. 3:**
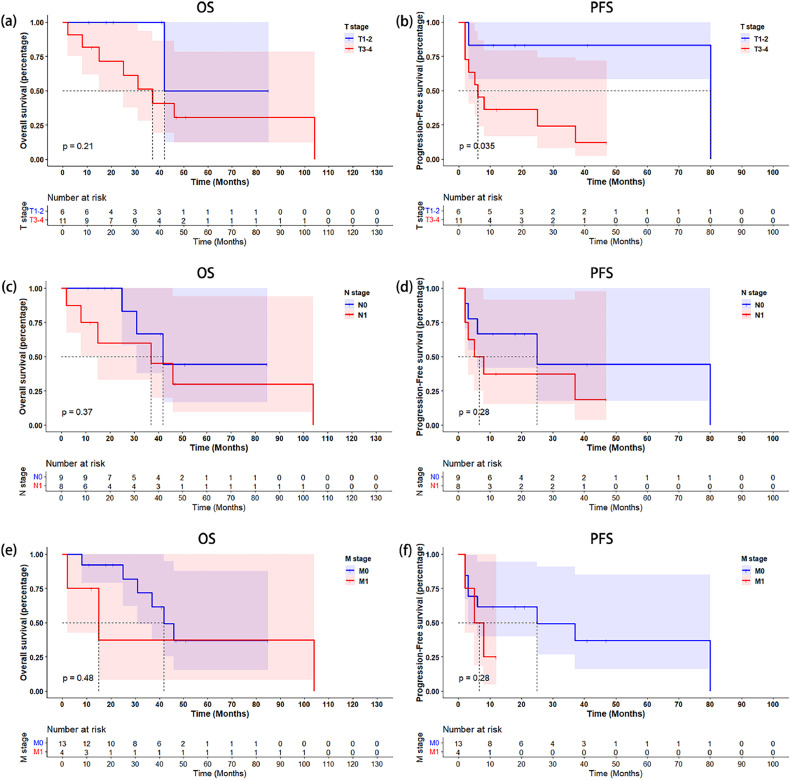
Kaplan-Meier plots illustrating OS and PFS in PUCP patients according to indicated subgroups. (a) OS according to T stage (T1–2 vs. T3–4). (b) PFS according to T stage (T1–2 vs. T3–4). (c) OS according to N stage (N0 vs. N1). (d) PFS according to N stage (N0 vs. N1). (e) OS according to M stage (M0 vs. M1). (f) PFS according to M stage (M0 vs. M1). OS, overall survival; PFS, progression-free survival; PUCP, primary urothelial carcinoma of the prostate.

Follow-up information was not available for one patient. In the remaining 17 patients, the median OS was 42 months (95 % CI: 28.74–55.26) (Fig. 2a), and the median PFS 25 months (95 % CI: 0–55.63) (Fig. 2b). According to log-rank tests, the OS rate did not differ significantly between patients with T1–2 and T3–4 disease (p=0.21) (Fig. 3a). However, the PFS rate was significantly higher in T1–2 patients than in T3–4 patients (p=0.035) (Fig. 3b). Further, OS and PFS did not differ significantly between patients with and without regional lymph node metastases (p=0.37 and p=0.28, respectively) (Fig. 3c,d), or between patients with and without distant metastases (p=0.48 and p=0.28, respectively) (Fig. 3e,f). Eight of eighteen patients in our cohort received chemotherapy solely or chemotherapy combined with other treatment, and two patients did not have information of progression to analyze objective response rate. The objective response rate with chemotherapy at three months after diagnosis was 66.7 % (4/6), and was 33.3 % (2/6) at six months after diagnosis.

## Discussion

Although the incidence of involvement of the prostate by urothelial carcinoma is reportedly between 12 % and 48 %, PUCP is rare [11], [12], [13]. Because of the low sensitivity and specificity, both underdiagnosis and misdiagnosis of PUCP is common. In our study, over half of patients had T4 tumors or lymph nodule metastasis, and nearly a third of patients had distant metastasis, at the time of diagnosis. One review of six patients with PUCP found distant metastases in all six, which demonstrates the hidden nature and extreme aggressiveness of PUCP [14]. Two case reports have suggested that PUCP patients are nearly 10 years younger than those with prostatic adenocarcinoma at the time of diagnosis [10,15]. However, our patients’ mean age was 72.4 years, which differs little from that of patients with glandular adenocarcinoma of the prostate. The average age of 69.1 years at diagnosis in one reported PCUP cohort is consistent with our findings [14].

Most of the symptoms of PUCP are non-specific lower urinary tract symptoms, including dysuria, urinary frequency, nocturia, and pain on urination [10,[15], [16], [17], [18]]. Gross hematuria can also occur [2,9,16,17]. In our cohort, the most common symptoms of PUCP were dysuria and urinary frequency (77.8 %), followed by gross hematuria (38.9 %) and pain on urination (33.3 %). These symptoms are similar to those of benign prostatic hyperplasia or prostatic adenocarcinoma, making the differential diagnosis challenging. Although hard tumor nodules were discovered on DRE in four patients, this did not enable distinguishing between PUCP and prostatic adenocarcinoma. Additionally, several particular symptoms have been reported in some PUCP patients. Zhang *et al*. reported a patient with PUCP with sustained fever for over one month [2]. Wadhwa *et al*. reported a patient with atypical rectal bleeding and an overlying rectal ulcer detected by DRE [8]. Further, a patient with PUCP reportedly developed a firm, nontender, metastatic nodule in the abdominal wall adjacent to his umbilicus during hospitalization [19].

Although only one third of our study patients had gross hematuria, erythrocytes were detected in 88.2 % of patients on urinalysis. In addition, 70.6 % and 88.2 % of our study patients were also positive for leukocytes and protein, respectively, on urinalysis. It seems that abnormal findings on urinalysis are a universal characteristic of PUCP. Maruyama et al. have also reported a PUCP patient who was positive for leukocytes, erythrocytes, and protein on urinalysis [18].

As for imaging findings, the reported diagnostic sensitivity of imaging in PUCP patients is inconsistent between different modalities. Using urological ultrasound, Yang et al. identified enlarged, irregularly shaped prostate glands suggestive of neoplasm [17], whereas Tan et al. only found homogeneous enlarged prostates by transrectal ultrasonography [9]. In another study, tumor nodules were not identified by transrectal ultrasonography, but enhanced CT showed patchy hypodensity [8]. In our cohort, B-ultrasound and enhanced CT detected abnormalities in 55.6 % and 50 % of patients, respectively, which also shows that these two diagnostic modalities are insensitive. MRI is the most strongly recommended radiological examination for prostatic adenocarcinoma, having good sensitivity for detection of this condition, especially for tumors that are over 10 mm in diameter [20], [21], [22]. In our series, abnormalities suggestive of tumors were found by MRI in 88.9 % of PUCP patients. Other studies of PUCP have found hypointense masses on T2-weighted images and high signal nodules on diffusion-weighted images on MRI [2,17,18,23]. One recently published article stated that both the prostate tumor and its metastases showed intense fluorodeoxyglucose uptake on positron emission tomography-CT [23]. However, these lesions were indistinguishable from prostatic adenocarcinoma. The lack of specificity of radiological features makes it difficult to distinguish PUCP from other types of prostate cancer.

The tumor marker PSA is of significant diagnostic value for cancer detection and has revolutionized diagnosis of prostatic adenocarcinoma [24,25]. In the case of PUCP, the findings are conflicting. Some studies have reported that PUCP patients have normal serum PSA concentrations [2,10,15,16,18,23], whereas others have found high serum PSA concentrations in patients with PUCP [8,9,17,19]. Only two (11.1 %) patients in our study had high serum total PSA concentrations and no patient had elevated PSA density, which is remarkably different from the findings in patients with prostatic adenocarcinoma. Besides, Sołek *et al*. identified a PUCP patient with high β-human chorionic gonadotropin concentrations [16]. Dong et al. reported high concentrations of carcinoembryonic antigen in one case [23]. Even though we discovered high serum total PSA concentrations in two patients, no patient showed immunohistochemical positivity for PSA, prostate specific acid phosphatase, or prostate-specific membrane antigen. Other researchers have also generally found negativity for PSA and P501s in samples of PUCP [26,27]. Rather, we found that immunohistochemistry for CK7, CK20, GATA-3, and P63 was more likely to be positive. Although PSA has significant value in pathological diagnosis, there are a variety of non-specific markers for urothelial carcinoma. GATA-3 is a urothelial marker that can differentiate urothelial carcinoma from prostatic adenocarcinoma [28]. P63 and HMWCK, including 34βE12, have also been shown to be markers of urothelial carcinoma [7,26]. Fichtenbaum *et al*. have reported that CK5/6 and double-stained CK7/CK5 can discriminate between urothelial carcinoma *in situ* and invasive urothelial cancer in the prostate [29]. Our findings indicate that PUCP patients are prone to have negative PSA and positive P63, GATA-3, CK7, and CK20 on immunohistochemistry and that needle biopsy of the prostate is necessary.

Because no standard treatment has yet been established, there are multiple treatment strategies for PUCP. In our series, radical cystectomy was the most commonly performed surgical procedure, and gemcitabine plus cisplatin combined with radiotherapy the most commonly administered systematic therapy, these approaches being quite different to those used for prostatic adenocarcinoma but similar to those used for urothelial bladder cancer. Considering all our patients had histologically proven high-grade urothelial carcinoma, conservative treatment was contraindicated. Some urologists propose TURP to the bladder neck to remove all gross tumor, followed by BCG instillation to treat any localized residual PUCP [30,31]. ICUD-EAU International Consultation recommended that radical cystectomy should be performed in patients with early stage PUCP and gemcitabine combined with cisplatin should be the first-line chemotherapy regimen for advanced-stage PUCP [32].

The 10-year OS rate of patients with typical prostatic adenocarcinoma is over 70 % according to a recent large study [33]. However, in one study of only six patients with PUCP, the median OS was only 4.6 months [14]. In our cohort, the median OS and PFS were 42 months and 25 months, respectively, indicating that the prognosis is worse than that of prostatic adenocarcinoma. However, patients in our cohort had significantly prolonged OS and PFS compared with the six PUCP patients Mallén et al. reported [14]. It should be noticed that only two of six patients received radical cystectomy in Mallén's cohort, and others only received radical prostatectomy, palliative TURP, or BCG instillation. The only one patient who received radical prostatectomy in our cohort also progressed 2 months later and died only 8 months after diagnosis. We hypothesized that the application of radical cystectomy and chemotherapy of gemcitabine combined with cisplatin prolonged the prognosis of our patients. Besides, this article was published nearly 20 years ago. The more advanced surgical technology and nursing level might also bring survival advantages for our patients. Cancer is prone to develop at the junction between the urothelial and columnar epithelium in the prostatic ducts; thus, PUCP may originate from prostatic duct urothelium [34]. Unlike the bladder urothelium, the prostatic duct urothelium lacks a lamina propria, facilitating penetration of the basal membrane and invasion of the prostatic stroma by tumor [35]. Our TNM subgroup analyses revealed only that the PFS rate was significantly higher in T1–2 patients than in T3–4 patients (p=0.035). We found no significant differences in OS between any TNM subgroups or in PFS in patients with or without lymph node and distant metastases, possibly because of our small sample size. Liedberg *et al.* have proposed a unique staging system for PUCP that differs from the AJCC staging system for prostatic adenocarcinoma. One study of the impact of involvement of different sites in the prostate on the prognosis of urothelial carcinoma demonstrated that stromal involvement is associated with the worst outcomes [36]. More large studies should be conducted to explore the proposed unique staging system for PUCP.

As far as we know, this is the first multi-center study to explore the features, treatment, and outcomes of PUCP. However, several limitations of this study need to be discussed. First, its retrospective design may have led to bias in data selection and incomplete follow-up information. Second, although this is the largest series of PUCP reported so far, the cohort size was still too small to allow extensive exploration of treatment protocols and prognostic factors. Third, due to the lack of information, we did not describe the genomic characteristics of PUCP. The unique genomics of PUCP and the comparison with prostatic adenocarcinoma should be further analyzed in the future. Finally, patients were collected from different institutions, potentially resulting in heterogeneity of the detection and diagnostic criteria of clinical characteristics. In the future, these should ideally be balanced and analyzed in larger, well-designed prospective studies of patients. Despite these restrictions, our findings could contribute to a better understanding of PUCP.

## Conclusions

PUCP, a rare but highly aggressive type of prostate cancer, should be considered in men with abnormalities on MRI and normal serum PSA concentrations. Typical immunohistochemistry findings are positivity for GATA-3 and P63, and negativity for PSA. Radical cystectomy, chemotherapy with gemcitabine and cisplatin, and radiotherapy can be effective. Higher T stage is a significant risk factor for PFS. Large, well-designed prospective studies should be performed in the future.

## Funding

This study was partly funded by the 10.13039/501100007129Natural Science Foundation of Shandong Province (ZR2021MH354), Medical and health research program of Qingdao (2021-WJZD170). The funders had no roles in study design, data collection and analysis, decision to publish, or preparation of the manuscript.

## CRediT authorship contribution statement

**Junjie Ji:** Data curation, Formal analysis, Methodology, Writing – original draft, Writing – review & editing. **Tian Liu:** Formal analysis, Methodology, Investigation, Writing – review & editing. **Yu Yao:** Software, Methodology. **Wen Liu:** Data curation, Investigation. **Hao Ning:** Data curation, Investigation. **Tongyu Wang:** Data curation, Visualization. **Guiming Zhang:** Conceptualization, Funding acquisition, Project administration, Methodology, Writing – review & editing.

## Declaration of Competing Interest

The authors declare that they have no known competing financial interests or personal relationships that could have appeared to influence the work reported in this paper.
